# The Association of Vitamin D Receptor Polymorphisms with COVID-19 Severity

**DOI:** 10.3390/nu16050727

**Published:** 2024-03-02

**Authors:** Nikolaos Tentolouris, Charoula Achilla, Ioanna A. Anastasiou, Ioanna Eleftheriadou, Anastasios Tentolouris, Dimitrios Basoulis, Ourania Kosta, Alexandros Lambropoulos, Maria P. Yavropoulou, Anthoula Chatzikyriakidou, Edward B. Jude

**Affiliations:** 1 1st Department of Propaedeutic and Internal Medicine, Laiko University Hospital, Medical School, National and Kapodistrian University of Athens, 115 27 Athens, Greece; anastasioui@med.uoa.gr (I.A.A.); joeleftheriadou@yahoo.com (I.E.); antentol@med.uoa.gr (A.T.); dimitris.bassoulis@gmail.com (D.B.); raniakwsta@yahoo.com (O.K.); myavropoulou@med.uoa.gr (M.P.Y.); 2 Laboratory of Medical Biology—Genetics, Faculty of Medicine, School of Health Sciences, Aristotle University of Thessaloniki, 541 24 Thessaloniki, Greece; cachilla@auth.gr (C.A.); lambrop@auth.gr (A.L.); chatzikyra@auth.gr (A.C.); 3 Department of Diabetes and Endocrinology, Tameside and Glossop Integrated Care NHS Foundation Trust, Ashton-under-Lyne OL6 9RW, UK; edward.jude@tgh.nhs.uk; 4 Division of Diabetes, Endocrinology and Gastroenterology, The University of Manchester, Manchester M13 9PL, UK; 5 Faculty of Science and Engineering, Manchester Metropolitan University, Manchester M15 6BH, UK

**Keywords:** COVID-19, vitamin D receptor polymorphisms, Greek population, severity

## Abstract

Background: Association studies of vitamin D receptor (VDR) polymorphisms with COVID-19 severity have produced inconsistent results in different populations. Herein we examined *VDR* gene polymorphisms in a Caucasian Greek cohort of COVID-19 patients. Methods: This was a case-control study in a tertiary university hospital in Greece including 137 COVID-19 patients with varying disease severities and 72 healthy individuals. In total 209 individuals were genotyped for the FokI (rs10735810), ApaI (rs7975232), TaqI (rs731236) and BsmI (rs1544410) single-nucleotide polymorphisms (SNP) of the *VDR* gene by polymerase chain reaction and restriction fragment length polymorphism analysis (PCR-RFLPs). Statistical analyses were performed to determine the association between genotype and disease severity, adjusting for various confounding factors. Results: Genotype distribution of the studied *VDR* SNPs in the control group was in Hardy–Weinberg equilibrium. The TaqI variant was differentially distributed between controls and COVID-19 patients according to the additive model (*p* = 0.009), and the CC genotype was significantly associated with an increased risk for severe COVID-19 according to the recessive model [OR: 2.52, 95%CI:1.2–5.29, *p* = 0.01]. Multivariate analysis demonstrated a robust association of COVID-19 severity and TaqI polymorphism in the recessive model even after adjusting for multiple confounders, including age, sex and CRP levels [Adj.OR:3.23, 95%CI:1.17–8.86, *p* = 0.023]. The distribution of FokI, ApaI and BsmI genotypes was similar between COVID-19 patients and controls. Conclusions: The CC genotype of TaqI polymorphism is significantly associated with an increased risk for severe COVID-19 independently of age, sex or degree of inflammation.

## 1. Introduction

Since its first outbreak in March 2020, acute infection of the respiratory tract caused by a novel coronavirus (severe acute respiratory syndrome coronavirus 2 [SARS-CoV-2]) has so far infected more than 771,407,825 people and led to 6,972,152 deaths (18 October 2023 [http://covid19.who.int/]). Although many patients infected with coronavirus disease (COVID-19) are asymptomatic or have mild symptoms [1], 10 to 15% present severe symptoms and may require invasive mechanical ventilation [2]. Several studies have shown a possible negative effect of vitamin D deficiency on both susceptibility to COVID-19 and severity of the disease [3]. Based mostly on observational studies, lower serum concentrations of 25-hydroxyvitamin D [25(OH)D] were associated with greater mortality, greater need for intensive care treatment and increased severity of illness in general [3]. However, the inability to adjust for confounding factors in these earlier studies and the fact that the sampling time in many of them differed between groups could hamper the validity of their results. 

Supplementation of vitamin D in hospitalized COVID-19 patients has demonstrated conflicting results. In a recent placebo-controlled intervention study in 240 hospitalized COVID-19 patients a bolus vitamin dose (200,000 IU) did not show beneficial effects in terms of length of stay (defined as the time from the date of randomization to hospital discharge), mortality during hospitalization, risk of admission to the intensive care unit and need for or duration of mechanical ventilation [4]. In this study, the mean baseline 25(OH)D concentration was 50 nmol/L, while 50% of the study cohort (57 patients in the vitamin D group and 58 patients in the placebo group) had vitamin D deficiency [4]. In a post-hoc analysis, supplementation for patients with 25(OH)D deficiency at baseline also did not have a significant impact on length of hospital stay or in-hospital mortality [4]. Conflicting results were demonstrated in a smaller-size pilot randomized non-placebo controlled open label study, where treatment with calcifediol or 25(OH)D in 50 hospitalized patients significantly reduced the need for intensive care unit admission [5]. In addition, large, randomized studies that studied the relationship of up to eight SNPs related to genes for vitamin D metabolism revealed that up to 4.3% of the variance in serum 25(OH)D levels also showed no causal relationship between genetically determined low 25(OH)D levels and the risk of COVID-19 susceptibility, severity or increased hospitalization [6,7]. Thus, the link between vitamin D status and the severity of COVID-19 remains largely unsettled. 

Vitamin D receptor (VDR) is widely expressed in different tissues regulating a variety of physiological actions. Several VDR gene polymorphisms have been identified in the last decades and have been associated with many diseases’ susceptibility. With respect to the immune system, different polymorphisms of the VDR gene, such as FokI, BsmI, ApaI, and TaqI, have been linked to tuberculosis [8,9,10] and severe respiratory syncytial virus (RSV) bronchiolitis [11]. 

To date, several studies have investigated the role of VDR gene polymorphisms in the outcome of COVID-19, showing inconsistent results in different ethnicities and geographical regions and in regard to various vitamin D levels [12,13,14,15,16]. The aim of the present study was to evaluate the association between the VDR gene polymorphisms FokI (rs10735810), ApaI (rs7975232), TaqI (rs731236) and BsmI (rs1544410) and the severity of COVID-19 in the setting of a single tertiary centre during the period of the COVID-19 pandemic and irrespective of vitamin D levels in the serum.

## 2. Patients and Methods

### 2.1. Participants 

**Figure 1:**
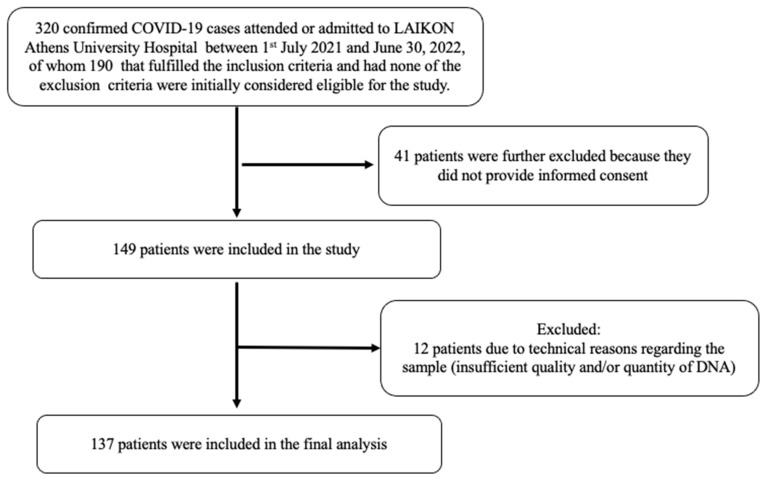
Flow diagram of included COVID-19 patients.

This was a single-center case-control study. Consecutive patients attended or admitted to the Laiko University Hospital of Athens in Greece from 1 July 2021 to 30 June 2022 were initially screened for eligibility (Figure 1). Inclusion criteria required that participants were adults and were able to read and understand the informed consent form. We excluded patients (i) with malignancy, (ii) receiving immunosuppressive therapy, (iii) on hemodialysis or (iv) with advanced liver disease. A detailed medical history was obtained and data regarding demographics and comorbidities [chronic obstructive pulmonary disease (COPD), coronary artery disease (CAD), stroke, hypertension, and diabetes mellitus (DM)] as well as smoking habits were recorded in compliance with the General Data Protection Regulation (GDPR) policy of the hospital. Body weight and height were measured in participants who were able to stand and body mass index (BMI) was calculated, while the reported weight and height from patients who could not stand (n = 12 with severe COVID-19) were not used in the study. In addition, we collected data for the outcomes of the participants from the hospital records and by telephone interview 1 month after recruitment for those hospitalized, and by telephone interview 1 month after recruitment for those with mild symptoms.

Laboratory-confirmed SARS-CoV-2 infection was defined as a case testing positive through real-time reverse-transcriptase polymerase chain reaction (RT-PCR, SARS-CoV-2 Variants ELITe MGB^®^ Kit, ELITechGroup; Puteaux, France). Apparently healthy volunteers were recruited from the outpatient clinics of the same hospital during the same time period and served as controls. Only asymptomatic volunteers with negative RT-PCR test for SARS-CoV-2 and without recent contact with a COVID-19 case at the time of blood sampling were included. 

The severity of COVID-19 in patients was classified according to World Health Organization (WHO) criteria. Mild illness: individuals who have any of the various signs and symptoms of COVID-19 (e.g., fever, cough, sore throat, malaise, headache, muscle pain, nausea, vomiting, diarrhoea, loss of taste and smell) but who do not have shortness of breath or abnormal chest imaging. Severe illness: individuals who have SpO_2_ < 94% on room air at sea level, a ratio of arterial partial pressure of oxygen to fraction of inspired oxygen (PaO_2_/FiO_2_) < 300 mm Hg, a respiratory rate > 30 breaths/min, or lung infiltrates >50% [17]. 

All participants signed an informed consent form and the procedures followed were in accordance with the 1975/83 Declaration of Helsinki. The protocol was approved by the Ethics Committee of the Laiko General Hospital, Athens, Greece (EC: 444/28-06-2021, 28 June 2021). 

### 2.2. Sampling Procedure and Measurements

#### 2.2.1. Biochemical Analyses

Blood samples were obtained from patients and controls immediately upon recruitment at an unscheduled timepoint and were centrifuged, and serum aliquots were stored at −20 °C until further analysis.

Full blood count analysis was performed on a SYSMEX XS-1000i (SYSMEX EUROPE GMBH, Norderstedt, Germany) automatic analyzer. Biochemistry measurement (serum creatinine, lactate dehydrogenase [LDH], and ferritin) was obtained by colorimetry. Measurement of d-dimers was carried out with a quantitative enzyme-linked immunoassay automated on a VIDAS immunoanalyzer (VIDAS d-dimer^®^, Bio-Mérieux, Lyon, France). CRP was measured with an immunoturbidimetric agglutination assay using a Roche 917 analyzer (Roche, Tina-quant^®^; Mannheim, Germany). HbA1c was measured using a latex–immunoagglutination–inhibition method (Bayer HealthCare LLC, Elkhart, IN, USA) with a non-diabetic range of 4.0–6.0% (20–42 mmol/mol). Serum 25(OH)D concentrations were measured using an electrochemiluminescence immunoassay (ECLIA) on a COBAS analyzer (Cobas 6000, Roche Diagnostics International Ltd., CH-6343 Rotkreuz, Switzerland). Serum 25(OH)D levels < 10 ng/mL were considered deficiency, 10–20 ng/mL inadequacy, and 20–40 mg/mL sufficiency [18]. 

#### 2.2.2. Genetic Analysis

Genomic DNA of 209 individuals was extracted from peripheral blood lymphocytes using the QIAamp DNA Mini Kit (Qiagen, Hilden, Germany). VDR FokI (rs10735810), ApaI (rs7975232), TaqI (rs731236) and BsmI (rs1544410) polymorphisms were studied by polymerase chain reaction-restriction fragment length polymorphism (PCR-RFLP) assay, as previously described [19]. All samples were run twice. 

### 2.3. Statistics

The Shapiro–Wilk test was used to assess for normality of distributions, and the data are presented as mean ± standard deviation (SD) or median (25, 75 percentile), as applicable. Student’s *t*-test for independent samples or the Mann–Whitney U-test was used for comparisons of continuous variables between patients and controls, as applicable. For comparison of continuous variables among more than 2 groups, we used one-way ANOVA followed by Bonferroni’s multiple comparisons test, or the Kruskal–Wallis test followed by Dunn’s multiple comparisons test for non-normally distributed variables. Comparisons between groups for non-parametric data were performed using the Mann–Whitney U test. 

Because the number of the participants with CAD and stroke were small, they were included together with hypertension patients in one group as cardiovascular disease (CVD) in logistic regression analysis.

Categorical variables were analyzed with Chi-square and Fisher’s exact tests. In addition, as no significant differences were found regarding FokI, ApaI, TaqI and BsmI polymorphisms between controls and COVID-19 positive participants with mild symptoms (Supplementary Table S1), we combined them into one group in logistic regression analysis.

Pearson’s chi-square test was used to test for differences in polymorphism distribution between COVID-19 patients and controls under the six models of genetic association, which are homozygote, heterozygote, dominant, recessive, allelic, and additive. When expected values were less than 5, Fisher’s exact test was used instead. Furthermore, the odds ratio (OR) with a confidence interval (CI) of 95% was calculated. Pearson’s chi-square test was also used to examine possible deviations of genotype distributions from the Hardy–Weinberg equilibrium (HWE) in the control group.

Univariable linear regression analysis was used to test for associations of severe COVID-19 outcome with the tested parameters. Multivariable logistic regression analysis was performed to search for independent associations between severe COVID-19 outcome and VDR polymorphisms after adjusting for the effects of the variables that were found to be significantly associated with the outcome in the univariable model.

All *p*-values are two-sided and a value of *p* < 0.05 was considered statistically significant. Statistical analysis was performed using IBM SPSS Statistics for Windows, Version 26 (IBM SPSS Statistics for Windows, IBM Corporation, Armonk, NY, USA) and GraphPad Prism for Windows, Version 7 (GraphPad Software, San Diego, CA, USA).

## 3. Results

A total of 137 patients with COVID-19, of which 51 were with mild symptoms and 86 were hospitalised for severe disease, and 72 controls were included in the analysis. Demographic and clinical characteristics, as well as standard laboratory work, are depicted in Table 1. The number of males was higher among severe COVID-19 patients, whereas age and BMI were similar between mild and severe COVID-19 patients and controls. Vitamin D concentration and status, white blood cell count, hemoglobin, and creatinine did not differ significantly among the three groups. DM and hypertension were the most common comorbidities in COVID-19 patients. No significant differences were found regarding the presence of concomitant diseases, such as DM, COPD, stroke, CAD, hypertension, and smoking status between the three groups. As expected, the median CRP levels were almost 5-fold higher in patients with severe COVID 19 than in patients with mild disease and 20-fold higher than controls (Table 1).

During the first month of follow-up, none of the study participants with mild symptoms died or were admitted to the intensive care unit (ICU). Ten patients (11.6%) with severe COVID-19 died during hospitalization and six (7%) were admitted to the ICU. The median length of hospital stay was 9.0 (5.0, 13.0) days.

### Genetic Analysis

Four genetic variants in *VDR* (FokI, ApaI, TaqI and BsmI) were genotyped. The genotype distribution of all the SNPs was in Hardy–Weinberg equilibrium in the control group (Table 2). Comparing genotype distribution of the studied *VDR* SNPs between controls/mild symptoms and severe COVID-19 patients, a statistically significant difference was revealed in the case of TaqI polymorphism according to the additive model (TT vs. TC vs. CC, respectively *p* = 0.009). In addition, according to the recessive model (CC vs. TC + TT), the CC genotype of TaqI polymorphism was significantly associated with increased risk for severe COVID-19 [OR:2.52, 95%CI:1.2–5.29, *p* = 0.01] (Table 2). 

No significant association was found between FokI, ApaI or BsmI genotypes and COVID-19 (Supplementary Table S1).

Univariable logistic regression analysis showed that COVID-19 severity was also significantly associated with age, gender (female vs. male) [OR 0.331, 95% CI 0.185–0.595, *p* < 0.001], serum CRP levels, lymphocyte number LDH concentrations, serum ferritin levels and D-dimers, as shown in Table 3. No significant associations were found between COVID-19 severity and BMI, smoking habits, 25(OH)D levels, DM, COPD or CVD (Table 3). Multivariable logistic regression analysis was then performed to further analyze the association between COVID-19 severity and TaqI polymorphism after adjusting for significant confounders (Table 3). We demonstrate that COVID-19 severity was significantly associated with TaqI polymorphism in the recessive model (CC vs. TC + TT) independent of age, gender, serum CRP levels, absolute lymphocyte number, LDH, ferritin levels and D-dimers [adj. OR 3.234, 95% CI 1.179–8.869, *p* = 0.023].

Notably, in our multivariable logistic regression model, age, male gender, low lymphocyte number, serum CRP, ferritin levels and LDH also demonstrated significant and independent associations with COVID-19 severity (Table 3).

## 4. Conclusions

Vitamin D receptor is expressed in a wide variety of immune cells, such as T and B lymphocytes, macrophages and monocytes [20], and plays a critical role in the regulation of the immune system (reviewed in [21]). As such, the association of *VDR* polymorphisms with either viral [22] or microbial infections [8,23] has been an area of great interest in scientific research. 

In our study, we studied the associations of four genetic *VDR* variants, namely FokI, ApaI, TaqI and BsmI, with COVID-19 severity. We showed that the CC genotype of TaqI polymorphism is significantly associated with increased risk for severe COVID-19 independently of age, sex and degree of inflammation, while no significant association was found for FokI, ApaI or BsmI genotypes.

The TaqI polymorphism is located on exon 9 and, by itself or acting as an association signal for a nearby mutation due to linkage disequilibrium, could affect VDR’s mRNA stability, leading to an alteration in its protein expression levels [24]. Previously, the *VDR* TaqI variant has been linked to greater risks of acute lower respiratory infection in early childhood [25], and of tuberculosis in South and West Asians [26].

In a recent study involving 500 adult Iranian COVID-19 patients with varying severities, *VDR* TaqI polymorphisms were not associated with clinical manifestation or severity of COVID-19 [12]. In this study, which did not include an uninfected control group, other *VDR* gene polymorphisms were significantly associated with clinical manifestations and various comorbidities in patients with severe/critical COVID-19 [12]. In another study of 517 Portuguese patients with COVID-19, among the 18 genetic variants associated with vitamin D pathways that were studied, polymorphism rs2282679 in the Vitamin D-binding protein (DBP) encoded by the *GC* gene was related to infection severity, while the authors did not find an association with the studied polymorphisms in the *VDR* gene [13]. The TaqI polymorphism of the *VDR* gene was also evaluated in a small cohort of Cuban citizens with COVID-19. In this small study, patients with the tt genotype were more likely to develop symptomatic [OR = 2.081, 95% CI: 0.243–17.842] and more severe forms [OR = 1.200, 95% CI: 0.217–6.638] of the disease [14].

These studies, however, did not include a control group of uninfected individuals, thus limiting the robustness and validity of those early findings by failing to assess causality, generalize results, or reduce the risk of bias within the cohort. In two recent case-control studies, TaqI and FokI polymorphisms were associated with increased susceptibility to and severity of the disease in two populations of different geographical origin: Turkey [15] and Cyprus [16]. In the study from Turkey, presenting a cohort of approximately 300 COVID-19 patients, the TaqI TT genotype was associated with a poor prognosis and increased admission to ICU [15]. In this study, the FokI f allele affected the prognosis of patients, while the ApaI aa genotype was associated with higher mortality rates [15]. In the cohort of 600 individuals (of whom 300 were SARS-CoV-2 infection positive) from Cyprus, the FokI T and TaqI C alleles were more frequently observed in SARS-CoV-2-infected patients compared to the control group (*p* < 0.005), pointing to a linkage disequilibrium between these VDR variants [16]. However, this study could not confirm the potential association of FokI T and TaqI C polymorphisms with severity of COVID-19 due to lack of access to the patients’ clinical data [16]. 

The varying associations between gene polymorphisms in *VDR* and susceptibility to infection across different geographic populations can be attributed to several factors. Firstly, genetic diversity among populations results in distinct alleles and haplotypes in the *VDR* gene, which can impact immune responses to infections differently. Additionally, environmental factors, such as sunlight exposure and diet, play a crucial role in vitamin D synthesis and metabolism, affecting the activity of the *VDR* gene. Differences in sunlight availability and dietary habits across regions can thus lead to disparate associations between *VDR* polymorphisms and infection susceptibility. Finally, interethnic differences in the prevalence of infectious diseases and pathogen exposure patterns can influence the selective pressures acting on *VDR* and other immune-related genes among populations. These factors collectively highlight the importance of considering population-specific factors when studying the association between *VDR* polymorphisms and infection susceptibility [27,28].

It is worth mentioning that our study has some limitations. First, this was a relatively small-sized study restricted to the broader urban area of Attiki in Greece. Second, no data were available regarding medication of COVID-19 patients in order to adjust for possible confounders in the outcomes of severe COVID-19. Third, the genetic analysis was restricted to four genetic variants of *VDR,* and no other genes that regulate or are related to vitamin D metabolism were assessed. Nevertheless, to the best of our knowledge, this is the first case-control study evaluating the association between VDR polymorphisms and severity of COVID-19 in the Greek population. Our findings are in line with other studies showing a positive association of the TaqI C allele with COVID-19 morbidity [16,29]. 

COVID-19 remains an important public health concern worldwide, and studies that investigate the impact of VDR polymorphisms in the severity of the disease across different populations are needed in order to pave the way towards a more patient-centric and customized therapeutic approach for infected individuals. Ongoing research on the genetic factors that may contribute to the severity of the disease remains an unmet need towards optimal risk stratification and targeted interventions.

## Figures and Tables

**Table 1:** Demographic and clinical characteristics and laboratory parameters of the study participants classified by study group.

Parameters	Controls (Group 1) n = 72	Mild COVID-19 (Group 2) n = 51	Severe COVID-19 (Group 3) n = 86	*p*-Value All Groups	*p*-Value 1 vs. 2	*p*-Value 1 vs. 3	*p*-Value 2 vs. 3
Gender (males), n (%)	30 (41.7%)	25 (49%)	61 (70.9%)	0.001 ^ǂ^	0.419 ^×^	<0.001 ^×^	0.010 ^×^
Age (years)	56.0 ± 11.4	56.51 ± 16.6	60.5 ± 12.5	0.072 *	-	-	-
Weight (kg)	81.76 ± 19.8	78.99 ± 15.2	84.33 ± 17.2	0.236 *	-	-	-
BMI (kg/m^2^)	28.9 ± 5.4	27.6 ± 4.4	28.6 ± 6.4	0.429 *	-	-	-
Lymphocytes, n	2200 (1760, 2615)	1360 (705, 2250)	900 (645, 1245)	<0.001 ^	<0.001 ^†^	<0.001 ^†^	0.003 ^†^
WBC, n/μL	7020 (6205, 8297.5)	6215 (4650, 9245)	6270 (4710, 9115)	0.219 ^			
Serum 25(OH)D (ng/mL)	28 (20.0, 33.7)	25.20 (16.6, 37.0)	23.05 (13.2, 30.0)	0.120 ^			
Vitamin D status				0.220 ^ǂ^			
Deficiency, n (%)	1 (1.4)	2 (4.0)	5 (5.8)				
Inadequacy, n (%)	12 (16.7)	4 (7.8)	17 (19.8)				
Sufficiency, n (%)	59 (81.9)	45 (88.2)	64 (74.4)				
Hb (g/dL)	13.7 ± 1.5	12.9 ± 2.1	13.3 ± 3.9	0.286 *			
Serum Creatinine (mg/dL)	0.84 ± 0.36	0.87 ± 0.4	1.5 ± 3.9	0.125 *			
LDH (U/L)	180.5 (153.8, 205.8)	215 (179.8, 297.0)	312.5 (222.8, 415.3)	<0.001 ^	<0.001 ^†^	<0.001 ^†^	<0.001 ^†^
Serum CRP (mg/L)	2 (0.85, 4.6)	9.2 (3, 45.4)	43 (12.1, 91)	<0.001 ^	<0.001 ^†^	<0.001 ^†^	<0.001 ^†^
Ferritin (ng/mL)	66.5 (35.0, 97.0)	211.0 (54.3, 443.5)	528.5 (260.3, 1110.3)	<0.001 ^	<0.001 ^†^	<0.001 ^†^	<0.001 ^†^
D-dimers (μg/mL)	0.5 (0.3-0.6)	0.6 (0.3, 1.4)	0.9 (0.5, 3.7)	<0.001	0.065 ^†^	<0.001 ^†^	0.020 ^†^
HbA1c (%)	6.3 (5.0, 12.7)	6.1 (5.1, 7.9)	7.6 (5.5, 12.7)	0.012 ^	0.327 ^†^	0.005 ^†^	0.023 ^†^
DM, n (%)	22 (30.6)	9 (17.6)	18 (20.9)	0.193 ^ǂ^		-	-
COPD, n (%)	0 (0)	2 (3.9)	2 (2.3)	0.273 ^ǂ^		-	-
Stroke n (%)	0 (0)	3 (5.9)	4 (4.7)	0.141 ^ǂ^			
CAD, n (%)	8 (11.1)	4 (7.8)	5 (5.8)	0.477 ^ǂ^			
Hypertension, n (%)	22 (30.6)	14 (27.5)	30 (34.9)	0.647 ^ǂ^			
Current smokers, n (%)	13 (18.1)	10 (19.6)	8 (9.3)	0.166 ^ǂ^			

Data are shown as mean ± SD, or median (25, 75 percentile) as applicable. Categorical data are shown as absolute number (valid percentage). * *p*-values for comparisons between all three groups by one-way analysis of variance (ANOVA). ^ *p*-values for comparisons between all three groups by Kruskal–Wallis H test. ^ǂ^ *p*-values for comparisons between all three groups by Chi-square test. ^†^ *p*-values for comparisons between pairs of groups (1 vs. 2, 1 vs. 3, 2 vs. 3) by Mann–Whitney U test. ^×^ *p*-values for comparisons between pairs of groups (1 vs. 2, 1 vs. 3, 2 vs. 3) by Chi-square test. BMI: body mass index, WBC: white blood cells, 25(OH)D: 25-hydroxyvitamin D, Hb: hemoglobin, LDH: lactate dehydrogenase, CRP: C-reactive protein, HbA1c: glycated hemoglobin A1c, DM: diabetes mellitus, COPD: chronic obstructive pulmonary disease, CAD: coronary artery disease.

**Table 2:** The association of TaqI (rs731236) polymorphism with COVID-19 severity.

SNP Genotypes	Controls/Mild Symptoms	Severe Symptoms	Analysis Model	OR (95%CI)	*p*-Value	HWE in Control Group (*p*-Value)
TT	43	35	Additive (TT vs. TC vs. CC)		0.009	0.35
TC	66	30				
CC	14	21				
TT	43	35	Homozygous (CC vs. TT)	1.84 (0.82–4.14)	0.14	
CC	14	21				
TT	43	35	Heterozygous (TC vs. TT)	0.56 (0.3–1.04)	0.07	
TC	66	30				
TT	43	35	Dominant (TC + CC vs. TT)	0.78 (0.44–1.38)	0.4	
TC + CC	80	51				
TC + TT	109	65	Recessive (CC vs. TC + TT)	2.52 (1.2–5.29)	0.01	
CC	14	21				
T	152	100	Allelic (C vs. T)	0.86 (0.58–1.28)	0.45	
C	94	72				

*p*-values are for comparisons between the two groups by Chi-square test. SNP, single nucleotide polymorphism, OR: odds ratio, CI: confidence interval, HWE: Hardy-Weinberg Equilibrium.

**Table 3:** Univariable and multivariable analyses on the associations between the studied parameters and severe COVID-19.

Parameters	Univariable Analysis		Multivariable Analysis *	
	OR (95% CI)	*p*-Value	OR (95% CI)	*p*-Value
TaqI Recessive (CC vs. TC/TT)	2.52 (1.2–5.29)	0.01	3.234 (1.179–8.869)	0.023
Age (years)	1.025 (1.003–1.047)	0.024	1.028 (1.002–1.055)	0.038
Gender (female vs. male)	0.331 (0.185–0.595)	<0.001	0.257 (0.123–0.537)	
Serum CRP levels (mg/dL)	1.024 (1.014–1.34)	<0.001	1.021 (1.011–1.031)	<0.001
Lymphocytes (k/μL)	0.998 (0.998–0.999)	<0.001	0.998 (0.998–0.999)	<0.001
LDH (U/L)	1.012 (1.008–1.016)	<0.001	1.008 (1.003–1.013)	0.001
Ferritin (ng/mL)	1.003 (1.002–1.005)	<0.001	1.003 (1.002–1.004)	<0.001
D-dimers (μg/mL)	1.004 (1.000–1.009)	0.035	1.003 (0.999–1.007)	0.087
Serum 25(OH)D levels (ng/mL)	0.970 (0.939–1.002)	0.063	-	
DM (yes vs. no)	0.895 (0.643–1.248)	0.514	-	
CAD (yes vs. no)	0.853 (0.355–2.049)	0.722	-	
COPD (yes vs. no)	1.417 (0.196–10.258)	0.730	-	
Smoking (yes vs. no)	0.534 (0.223–1.278)	0.159	-	
BMI (kg/m^2^)	1.009 (0.960–1.060)	0.725	-	
Interaction between TaqI additive (TT vs. TC vs. CC) model and serum vitamin D levels	0.967 (0.879–1.064)	0.487	-	
Interaction between TaqI recessive (CC vs TC/TT) model and serum vitamin D levels	0.881 (0.739–1.049)	0.154	-	

* Parameters that were found to be significantly associated with COVID-19 severity in the univariable regression model were then added into the multivariable model to further analyze the association between COVID-19 severity and TaqI polymorphism. Chi-square test: 87.295, *p*-value < 0.001. OR, odds ratio; CI, confidence interval; BMI, body mass index; CRP, C-reactive protein; 25(OH)D, 25 hydroxyvitamin D; DM, diabetes mellitus; LDH, lactate dehydrogenase; CAD, coronary artery disease; COPD, chronic obstructive pulmonary disease. Variables that showed non-significant association with COVID-19 outcome in univariable analysis were not added to the multivariable logistic regression model.

## Data Availability

The data presented in this study are available on request from the corresponding author.

## Supplementary Materials

The following supporting information can be downloaded at: https://www.mdpi.com/article/10.3390/nu16050727/s1, Table S1: The association of FokI, ApaI, and BsmI polymorphisms with COVID-19 severity. *p* values are for the comparisons between the two groups by Chi-squared test. SNP, single nucleotide polymorphism, OR: odds ratio, CI: confidence interval, HWE: hardy-weinberg equilibrium.
