# Supplementary material for: The Association of Vitamin D Receptor Polymorphisms with COVID-19 Severity
Source: Nutrients. 2024 Mar 2;16(5):727. doi: 10.3390/nu16050727 (PMC10935253; doi:10.3390/nu16050727)
Supplement: Supplementary file 1 [file nutrients-16-00727-s001.zip › nutrients-2879966-supplementary.pdf]

Suppl. Table 1. The association of FokI, ApaI, and BsmI polymorphisms with COVID-19 severity

SNP Genotypes	Controls/ Mild symptoms	Severe Symptoms	Analysis Model	OR	95%CI	*p*-value	HWE in control group (*p*-value)
**FokI (rs2228570)**							
**CC**	58	41	Additive (CC *vs*. CT *vs*. TT)			0.47	0.72
**CT**	54	33					
**TT**	11	12					
**CC**	58	41	Homozygous (TT *vs*. CC)	1.54	0.62-3.84	0.35	
**TT**	11	12					
**CC**	58	41	Heterozygous (CT *vs*. CC)	0.86	0.48-1.56	0.63	
**CT**	54	33					
**CC**	58	41	Dominant (CT+TT *vs*. CC)	0.98	0.56-1.7	0.94	
**CT+TT**	65	45					
**CT+CC**	112	74	Recessive (TT *vs*. CT+CC)	1.65	0.69-3.94	0.26	
**TT**	11	12					
**C**	170	115	Allelic (T *vs*. C)	1.11	0.73-1.68	0.63	
**T**	76	57					
**ApaI (rs79752)**							
**TT**	44	35	Additive (TT *vs*. TG *vs*. GG)			0.53	0.13
**TG**	72	44					
**GG**	7	7					
**TT**	44	35	Homozygous (GG *vs*. TT)	1.26	0.4-3.92	0.69	
**GG**	7	7					
**TT**	44	35	Heterozygous (TG *vs*. TT)	0.77	0.43-1.37	0.37	
**TG**	72	44					
**TT**	44	35	Dominant (TG+GG *vs*. TT)	0.81	0.46-1.43	0.47	
**TG+GG**	79	51					
**TG+TT**	116	79	Recessive (GG *vs*. TG+TT)	1.47	0.5-4.35	0.49	
**GG**	7	7					
**T**	160	114	Allelic (G *vs*. T)	0.95	0.63-1.43	0.79	
**G**	86	58					
**BsmI (rs1544410)**							
**AA**	27	27	Additive (AA *vs*. AG *vs*. GG)			0.15	0.8
**AG**	66	35					
**GG**	30	24					
**AA**	27	27	Homozygous (GG *vs*. AA)	0.8	0.38-1.71	0.56	
**GG**	30	24					
**AA**	27	27	Heterozygous (AG *vs*. AA)	0.53	0.27-1.04	0.06	
**AG**	66	35					
**AA**	27	27	Dominant (AG+GG *vs*. AA)	0.61	0.33-1.15	0.13	
**AG+GG**	96	59					
**AG+AA**	93	62	Recessive (GG *vs*. AG+AA)	1.2	0.64-2.24	0.57	
**GG**	30	24					
**A**	120	89	Allelic (G *vs*. A)	0.89	0.6-1.31	0.55	
**G**	126	83					

P values are for the comparisons between the two groups by Chi-squared test.
SNP, single nucleotide polymorphism, OR: odds ratio, CI: confidence interval, HWE: hardy-weinberg equilibrium.
